# The Butterflies of Barro Colorado Island, Panama: Local Extinction since the 1930s

**DOI:** 10.1371/journal.pone.0136623

**Published:** 2015-08-25

**Authors:** Yves Basset, Héctor Barrios, Simon Segar, Robert B. Srygley, Annette Aiello, Andrew D. Warren, Francisco Delgado, James Coronado, Jorge Lezcano, Stephany Arizala, Marleny Rivera, Filonila Perez, Ricardo Bobadilla, Yacksecari Lopez, José Alejandro Ramirez

**Affiliations:** 1 Smithsonian Tropical Research Institute, Apartado 0843–03092, Panama City, Republic of Panama; 2 Faculty of Science, University of South Bohemia and Institute of Entomology, Biology Centre of Czech Academy of Sciences, Branišovská 31, 370 05, České Budějovice, Czech Republic; 3 Universidad de Panamá, Maestria de Entomologia, 080814, Panama City, Republic of Panama; 4 Northern Plains Agricultural Lab, USDA-Agricultural Research Service, 1500 N. Central Ave., Sidney, Montana, 59270, United States of America; 5 McGuire Center for Lepidoptera and Biodiversity, Florida Museum of Natural History, University of Florida, 3215 Hull Rd., P.O. Box 112710, Gainesville, Florida, 32611–2710, United States of America; 6 Universidad de Panamá, Centro Regional Universitario de Veraguas, Santiago, Republic of Panama; Consiglio Nazionale delle Ricerche (CNR), ITALY

## Abstract

Few data are available about the regional or local extinction of tropical butterfly species. When confirmed, local extinction was often due to the loss of host-plant species. We used published lists and recent monitoring programs to evaluate changes in butterfly composition on Barro Colorado Island (BCI, Panama) between an old (1923–1943) and a recent (1993–2013) period. Although 601 butterfly species have been recorded from BCI during the 1923–2013 period, we estimate that 390 species are currently breeding on the island, including 34 cryptic species, currently only known by their DNA Barcode Index Number. Twenty-three butterfly species that were considered abundant during the old period could not be collected during the recent period, despite a much higher sampling effort in recent times. We consider these species locally extinct from BCI and they conservatively represent 6% of the estimated local pool of resident species. Extinct species represent distant phylogenetic branches and several families. The butterfly traits most likely to influence the probability of extinction were host growth form, wing size and host specificity, independently of the phylogenetic relationships among butterfly species. On BCI, most likely candidates for extinction were small hesperiids feeding on herbs (35% of extinct species). However, contrary to our working hypothesis, extinction of these species on BCI cannot be attributed to loss of host plants. In most cases these host plants remain extant, but they probably subsist at lower or more fragmented densities. Coupled with low dispersal power, this reduced availability of host plants has probably caused the local extinction of some butterfly species. Many more bird than butterfly species have been lost from BCI recently, confirming that small preserves may be far more effective at conserving invertebrates than vertebrates and, therefore, should not necessarily be neglected from a conservation viewpoint.

## Introduction

Habitat degradation is the biggest current threat to tropical insects; however, the effects of climate change may be soon more pervasive [1]. Butterflies are frequently used as indicators of environmental disturbance or environmental change because they offer a number of logistical advantages over other potential indicator taxa [2–4]. Butterflies are more conspicuous than other insect groups and are active during the day, and many species can be identified in the field. Moreover, butterflies and their larvae play important roles in ecosystem functioning, including nutrient cycling and pollination, and should be studied as targets of conservation in their own right [5–6].

In temperate areas, there is strong evidence of recent local and regional extinction of several butterfly species as the result of habitat fragmentation and global climate change. Furthermore, butterfly extinction rates appear to be more rapid than those of birds and plants [7]. Unlike temperate areas, no long-term monitoring scheme for butterflies has been established in the tropics until recently [8]. Thus, in the absence of baseline data, the impact of climate or environmental changes on butterflies and other tropical insects is difficult to evaluate [5]. Indeed few data are available about the regional or local extinctions of tropical butterfly species [6, 9]. Regionally, Larsen [9] showed that 97% of forest butterfly species in West Africa were still present despite massive shrinkage of pristine forests. Conversely, at a rather smaller scale, 38% of butterfly species recently became extinct in Singapore, mainly due to the loss of host-plant species [10,11].

Assessment of recent extinction rates can be compiled only when older, baseline, data are available. This lack of data is often problematic for tropical insects, even for butterflies. For example, very few local butterfly lists exist for rainforest locations in Central America [12–16] and often they do not include the taxonomically challenging but exceptionally diverse families Hesperiidae and Lycaenidae [17] (for instance, these two families account for 39% of butterfly records in the forest of Barro Colorado Island in Panama [18]).

An important exception is Barro Colorado Island (BCI) in Panama, which is one of the best-studied rainforest sites in the tropics [19] and for which older butterfly data are available. Huntington (1932) [12] listed 267 butterfly species for BCI. Although his list is outdated taxonomically, it provides an excellent basis to detect butterfly extinction during the last 80 years on BCI. Furthermore, since Huntington’s study [12], knowledge on the butterflies of BCI increased steadily, including reports about the taxonomy of resident species [20–22], their abundance and seasonality [23–24], their flight and orientation [25–27], their dispersal abilities [28], their ecology and interactions with other species [29–30], and their speciation and hybridization processes [31–32]. This wealth of information may prove important for a sound interpretation of long-term changes in butterfly populations on BCI.

In 2008 the Arthropod Initiative of the Smithsonian Institution Center for Tropical Forest Science and Forest Global Earth Observatory (CTFS-ForestGEO) started monitoring butterflies in the long-term within and near the permanent botanical plot of CTFS on BCI. Methods and an overview of the most common butterfly species are detailed in [8]. This and earlier though still recent butterfly monitoring schemes on the island [30,33] produced a considerable sampling effort, thus providing the opportunity to evaluate whether all butterflies species present on BCI in the 1930’s [12] could still be collected on the island today (see methods). However, assessing which butterfly species may have gone extinct recently on BCI requires that checklists are revised with sound taxonomy.

Karr [34] discussed the local extinction of 50–60 breeding bird species on BCI and concluded that factors most likely to contribute to bird extinction include increased predation, small island size, and limited environmental heterogeneity. Drivers of extinction may be different for invertebrates. There is little doubt that habitat modification and loss greatly affects local butterfly richness in tropical rainforests [6]. For example, butterflies that use trees as larval hosts are more likely to be absent in logged forests, whereas butterflies using grasses and lianas as larval hosts are likely to be more abundant in these forests [35]. Extinction risks in butterflies appear to be correlated with larval utilization of certain plant growth types, adult habitat specialization, larger body size, and narrow geographic range [6,10]. Thus, larval host plants represent a crucial factor in assessing extinction risks in butterflies [11].

The aims of this study are fourfold: (1) to estimate total butterfly species richness on the island; (2) to provide baseline data for assessing long-term changes of butterflies on BCI in the future; (3) to assess the likelihood of local butterfly extinctions on BCI since the 1930’s; and (4) to identify possible species traits (accounting for butterfly phylogeny) likely to influence the probability of extinction. Our working hypothesis is that likely butterfly extinction on the island may be related to the loss or severe reduction of larval host-plant species [11]. To achieve (1) and (2) we established a revised checklist of the butterflies of BCI, including Hesperiidae and Lycaenidae, and employing a ‘DNA barcode’ approach [36] (see methods). To estimate (3) we compared the older and recent species compilations (see methods) and to perform (4) we compiled the ecological information associated with each butterfly species recorded from BCI.

## Material and Methods

### Study site

Barro Colorado Island (9.15°N, 79.85°W, 120–160m asl) in Panama is a biological reserve. It receives an annual average rainfall of 2631 mm, and has an annual average daily maximum air temperature of 28.5°C. The 50 ha CTFS plot is located in the center of the island. A detailed description of the setting and of the CTFS plot may be found elsewhere [37,38]. Circa 1880, about 45% of what is now the island was covered with old growth forest, whereas the rest represented shifting agriculture, with a probable mosaic of corn, upland rice and secondary forests that were about 20 years old [39–41] (S.J. Wright, pers. comm). Around 1910, the Chagres River was dammed to create the Panama Canal. Cerro Barro Colorado, cut off from the mainland by the rising water, became a 1,542 ha island (M. Solano, STRI GIS laboratory, pers. comm.). Circa 1920–1923, 96.7% of the island was covered with forests [40] but agricultural clearings still existed and gradually regenerated into secondary forest after BCI became a protected biological reserve in 1923 [40,41]. By 1930, forest (“primeval” and secondary forests) occupied 99.2% of the island’s area, the rest representing various clearings, including laboratory buildings and a small agricultural area used to grow plantains, yucca and fruits, which was maintained until the 1940s and then grew back to forest [40] (S.J. Wright, pers. comm). In the 1990’s small areas were cleared for additional laboratory buildings, including the former small agricultural area. Currently, the island is 100% forested, bar a few man-made clearings concentrated in the laboratory and housing area and one lighthouse clearing. Hence, recent changes in the BCI vegetation have been relatively few [41], but may nevertheless have affected its butterfly communities. As Enders [40] indicated: “although insignificant in area, these clearings are important as they are occupied by both plant and animal forms that do not occur in other areas.”

### Compilation of butterfly data

We used 12 datasets to compile records of butterflies (Hesperiidae & Papilionoidea) collected or observed on BCI (Table 1) and updated butterfly taxonomy following Lamas [42]. The datasets represent a mix of published lists, on-line databases, recent observations (rearing caterpillars) and monitoring projects (Table 1) [8,12,23,30,33,43–50]. The total 11,737 butterfly records were not distributed evenly across the years but rather emphasized active collecting in the 1930’s and recent monitoring in the 2000’s (S1 Fig). Our analyses aim at comparing the two richest periods with records, the 1923–1943 period and the 1993–2013 period, hereafter termed old and recent periods, respectively. As far as possible, we consider data that span several years for defining these periods, because insect populations may fluctuate markedly from year to year in Panama [30,51] or even become temporarily extinct [24]. Other studies documenting insect extinctions also considered 20 years with no recorded sightings as an adequate period after which a species can be considered as extinct [52]. A few isolated records were also extracted from specialized publications and those are listed in S1 Appendix.

**Table 1:** Datasets used to compile a list of butterfly species collected or observed on BCI, 1923–2013. The number of records (individuals) are indicated for the old and recent periods, as well as for the entire period of study.

Dataset	Description	Reference(s)	1923–1943	1993–2013	1923–2013
Bell	Published lists (Hesperiidae only)	[43,44]	178	-	178
Huntington	Published list	[12]	850	-	850
Sheldon	Published list	[45]	325	-	325
USNM	On-line database, National Museum of Natural History, Washington	[46]	-	-	4
MCZ	On-line database, Museum of Comparative Zoology, Cambridge	[47]	62	-	90
STRI	On-line database, Smithsonian Tropical Research Institute synoptic dry collection	[48]	-	10	57
Emmel	Published list	[23]	-	-	1585
Delgado	F. Delgado unpublished records of Gordon Small's specimens (*)	-	-	-	165
Aiello	A. Aiello's rearing records on BCI, 1977–2007	[49]	-	2	121
Srygley	R.B. Srygley butterfly monitoring on BCI, 1988–1990 and 2003	[30,33]	-	462	1160
Coley	P.D. Coley & T.A. Kursar rearing records on BCI, 1996–2005 (**)	[50]	-	37	37
CTFS	CTFS-ForestGEO Arthropod Initiative, monitoring 2008–2013	[8]	-	7161	7161
Varia	Various articles in specialized literature	See S1 Appendix	3	-	4
Total			1418	7672	11737

(*) Lycaenidae only. All records coded with year 1974, being the mid-point of 1962–1986, this period corresponding to the stay of G. Small in Panama.

(**) All records coded with year 2000, being the “mid-point” of 1996–2005.

The highest number of records was obtained from the compilation of Huntington [12] for the old period, whereas the CTFS monitoring [8] yielded most records for the recent period (Table 1). Most (95%) of the older records were obtained from years 1931–1933 (S1 Fig). The old published lists [12, 43–45] listed, for each species, independent records by date and sex, by compiling observations obtained from different collectors during different periods of the year. It is impossible to provide an estimate of sampling effort for the old period but it is rather low as compared to the recent period in terms of number of records (Table 1). The lists of Bell (1931, 1937) [43,44] focus on Hesperiidae but they do not over-represent this group when comparing data to the recent period, as sampling effort is higher and unbiased towards specific groups for the latter. There is no reason to believe that there was under-representation of particular taxonomic groups within the old period, with the exception of the night-flying Hedylidae, which were not recorded during that period, and are therefore not included in the present study. In a few cases (13 species, 4% of the number of species recorded during the old period), species were characterized as being “abundant” or “frequent” without mention of the number of individuals actually collected or observed. These records were scored as 10 individuals.

For the recent period the Srygley and CTFS monitoring (Table 1) were rather complementary, in terms of surveying different habitats. The former was performed by walking trails and searching gaps, including the “laboratory” gap. Surveying included both the dry and wet season and overall sampling effort amounted to 237 person-hours of observation. The latter used standardized Pollard walks to calculate indices of butterfly species abundance along 10 linear transects of 500m that were repeatedly sampled over a given time interval (30 min.) [53]. These transects were all located within and near the CTFS plot, in the shady understory of the forest. Each transect was surveyed as three replicates in each of four surveys encompassing dry and wet seasons. In total 30 transect-replicates were performed during a survey and 120 during a year (details in [8]; total 345 person-hours of observation or 345km of transects). CTFS and Srygley records also included opportunistic netting and collecting with traps baited with fruits on BCI. Voucher specimens were deposited into the collections of the CTFS-ForestGEO Arthropod Initiative at STRI.

### Species identification

Butterfly identifications dating from the old period were made by professional taxonomists using morphological data [12,43,44]. In the recent period, many of our identifications relied both on morphological and molecular data. For the monitoring projects (Srygley, CTFS: Table 1), reference collections were built before the onset of monitoring and used later to identify, whenever possible, butterflies in flight. However, an appreciable proportion of specimens sighted was also collected for verifying identifications. For the largest dataset, CTFS, 2,461 specimens out of 7,159 (34%) were pinned specimens. This represents about 1,000 specimens more than the total number of observations recorded during the old period (Table 1). These pinned specimens were first identified morphologically using [16,54–56], more specific publications when needed, and expert opinions (see Acknowledgments). Second, 1,328 CTFS pinned specimens were extracted one leg each, which were then processed at the Biodiversity Institute of Ontario (University of Guelph) using methods in [57,58] to obtain DNA Cytochrome c oxidase subunit I (COI, ‘DNA barcode’) sequences. We obtained 1,248 sequences out of these specimens, representing 17% of the total CTFS records. Molecular data were used to confirm identifications based on morphology and to examine the possibility that morphological uniformity might conceal cryptic species. All DNA sequences are available publicly on-line in the BOLD database (http://www.boldsystems.org/, project BCIBT) and in GenBank (http://www.ncbi.nlm.nih.gov/genbank/, GenBank accession numbers: KP848543—KP849461). All species delineated by molecular data (where COI divergence > 2% between species: [59]) are unambiguously referred to by their Barcode Index Number (BIN), which can be used as interim taxonomic nomenclature if needed [59]. Since it was not possible to examine the older specimens collected on BCI, deposited in various collections, we also searched the BOLD database to associate BINs to butterfly species not collected by CTFS during the recent period, and to be sure that they were distinct from BINs of recently collected specimens.

As indicated earlier, we used Lamas [42] to update the taxonomy of older records. Following Braby *et al*. [60], we refrained from using subspecies, since they are often inconsistently defined and they frequently fail to reflect distinct evolutionary units according to population genetic structure. Our only exception to this rule was when we encountered distinct BINs for subspecies and in this case only, we list them as separate entities, awaiting further taxonomic analyses. Morphospecies not yet formally described but with distinct BINs are termed “cryptic” species, even if in some cases they can be easily distinguished morphologically from other species. Interim names for cryptic species follow BOLD recommendations. Cryptic species were ignored in comparisons of old vs. recent periods because (a) they were not recognized during the old period; and (b) in most cases their abundance was lower than relevant sister species within the recent period.

### Phylogenetic relationships

Following extensive searches of the BOLD BCI Butterfly Data Base, the BOLD public records and GenBank we found barcodes from 451 of the 601 butterfly species recorded in the study for inclusion in our phylogenetic analysis. Given the scale of the analysis (both in terms of taxon number and taxonomic scale) and the fact that by definition within BIN variation rarely exceeds between BIN variation we decided to choose one exemplar per BIN. Our primary criterion was the length of the sequence read, subsequent decisions on inclusion were made arbitrarily. All data were downloaded from BOLD (including sequences mined from GenBank) either directly or by using the ‘read.BOLD’ function in the R package ‘Spider’ [61]. For species to be included in further analyses (see below) for which no barcodes existed (mostly ‘extinct’ species) we inserted an additional congeneric species as a replacement (S1 Appendix). This decision was made based on the taxonomic scale of the analysis and the reduction in statistical power caused by the omission of 11 taxa. To minimize the genetic distance between the original species and the replacement we chose congeners from the same region, making the assumption that these would be more closely related than, for example, Asian congeners. Using BEAST v2.1.0 [62] we estimated both topological relationships and branch lengths in millions of years. We found that setting strong node age and rate priors reduced the computational time needed for the BEAST 2.0 analyses to converge after burnin and allowed better exploration of the model parameters. Importantly it led to increased Effective Sample Size (ESS) for most parameters. Our phylogeny was constrained to match all of the inter-familial relationships that received high support (posterior probability support of one in all cases) by Heikkilä *et al*. [63]. Furthermore the relationships among subfamilies of Nymphalidae were constrained following Wahlberg and Wheat [64]. We used a secondary calibration point to estimate the node ages of our phylogeny, namely an exponential prior on the node age of the subfamily Nymphalinae with an offset of 60 MY and a mean of 15. This reflected the range of age estimates for the subfamily given by Wahlberg [65]. Furthermore, we used a relaxed lognormal clock with a ucld.mean of 0.0177 substitutions per million years (M parameter in real space) and log-normal distribution with an S parameter of 0.1 (in log-normal space). This gave a median rate of 0.0177 substitutions per million years with a 5% quantile of 0.0149 and a 95% quantile of 0.0208. This prior is based on the insect COI substitution rate taken from Papadopoulou *et al*. (2010) [66], but with wider bounds. Whilst a TIM2+I+G model was selected by jModelTest v2.1.6 [67] we selected a Hasegawa, Kishino and Yano (HKY) substitution model with a Gamma shape and a proportion of invariant sites (HKY+I+G). This was because the low cytosine (0.08) and guanine (0.01) base frequencies made any substitution rates involving these bases difficult to estimate, leading to poor mixing in parameters related to substitution rates and incomplete convergence, even over hundreds of millions of generations. We ran two MCMC chains of 80,000,000 generations which were sampled every 4,000 generations. These chains were re-sampled every 16,000 generations to yield a total of 10,000 trees which were used in combination for estimating the topology and node ages after removing a ‘burnin’ of 10% for each chain. We assessed the convergence of each statistic using Tracer v1.6 [68] to ensure that all Effective Sample Sizes (ESSs) were all over 200. We summarized the trees and median node ages using TreeAnnotator v2.1.2 [69].

### Statistical methods

We assigned each butterfly species recorded on BCI during the period 1923–2013 to one of the categories of “abundance status” listed in Table 2. These categories are used as a convenience to characterize our overall dataset and not all of them may have a biological significance. Most of these categories indicate the uncertainty of the status of species but the following categories are of special interest in the context of this study:

- Category 7: these species were well represented throughout 1923–2013 and can probably be considered as resident on BCI.
- Category 8: these species may include new colonizers/invasive species to the island or species with a recent high increase in population size. However, because sampling effort was much larger in the recent period than in the older one (as judged by the number of individuals recorded, Table 1), sound interpretation of these data is difficult.
- Category 9: these species may be considered as locally extinct from BCI, or at least with extremely low populations. Despite being collected with frequency during the old period (sum of individuals ≥ 10) and a considerably higher sampling effort during the recent period, they could not be located anymore. Note that all species included in Category 9 have a significant Fisher test (p < 0.0001) when comparing their species abundances in old vs. recent periods to that of all individuals recorded during these periods.
- Category 4: although these species were not collected during the recent period, their status is unclear because they were not abundant during the old period. However, adding the number of species in Categories 9 and 4 provides us with a maximum number of species that may be extinct on BCI.

**Table 2:** Categories of abundance status used to characterize each butterfly species recorded on BCI during the period 1923–2013, and denominations used in the context of this study. Ind. = individuals.

Category	Description	No. of species
1	Species currently only recognized with molecular data (BIN). These are denominated **cryptic species**.	27
2	Species described after 1943. **Recently described species.**	25
3	Species with unclear status (too few data): sum of ind. in old period + sum of ind. in recent period < 10 ind. and no sum in each period = 0. **Unclear status throughout the study period.**	63
4	Species not collected in 1993–2013 but of unclear status: sum of ind. in old period < 10 ind. and sum of ind. in recent period = 0. **Unclear status, not collected recently.**	132
5	Species not collected in 1923–1943 but of unclear status: sum of ind. in old period = 0 ind. and sum of ind. in recent period < 10. **Unclear status, not collected during the older period**.	170
6	Species only occurring between 1944 and 1992: sum of ind. both in old and recent periods = 0 but collected on BCI. **Unclear status, collected in-between the old and recent periods.**	71
7	Species well represented throughout 1923–2013: sum of ind. in old period + sum of ind. in recent period > = 10 ind. and sum of ind. in either period > 0. **Common species.**	70
8	Species only occurring during 1993–2013: sum of ind. in recent period ≥ 10 ind. and sum of ind. in old period = 0. **Settler species.**	20
9	Species only occurring during 1923–1943: sum of ind. in old period ≥ 10 ind. and sum of ind. in recent period = 0. **Extinct species.**	23

In order to anchor any estimate of butterfly extinction on BCI, we estimated the species richness of butterflies on the island by three separate methods. First, we estimated the total number of species likely to be present in the shady understory of BCI forests by randomizing the cumulative number of species collected/observed during 23 surveys performed during 2008–2013 by CTFS and calculating the Incidence Coverage-based Estimator (ICE) with EstimateS 8.20 [70]. Second, we calculated similarly the ICE with the number of individuals and species collected/observed with all data available for the recent period (1993–2013). To appreciate the theoretical taxonomic knowledge of the BCI butterfly fauna, we plotted the year of species description against the cumulative number of BCI species described. For cryptic species uniquely known by their BIN, we used the year in which the species was first collected as the year of description. Last, we fitted a logistic power regression to the cumulative number of individuals sequenced against the cumulative number of cryptic species discovered.

We used Nonmetric multidimensional scaling (NDMS) to compare the faunal composition of years on record for which at least 300 butterfly individuals were recorded (years 1932, 1968, 1988, 2003 and 2008 to 2013; matrix of 592 species x 10 years; Bray-Curtis distance). We used WinKyst 1.0 of the CANOCO package for these calculations [71]. We further performed multiple regressions with the scores of the years on Axes 1 and 2 of the NDMS ordination as dependent variables and variables Year and number of individuals collected (as proxy for sampling effort) as independent variables, in order to test the influence of time.

We tested for phylogenetic signal with regards to extinction status across the whole 451 taxon phylogeny, for all 151 members of the family Hesperiidae (12/23 extinct species were hesperiids), and for all 161 species of Nymphalidae (7/23 extinct species were nymphalids). Not only are these the two most species rich families in our data set with the highest numbers of extinct species, but they also diverge with respect to host use. Many members of the family Hesperiidae are often herb or palm feeders, whilst members of Nymphalidae feed across a wider range of plants [72]. We predict that extinction will be more clumped in hesperiids, that are less capable of utilizing alternative hosts and likely have more conserved patterns of host use than nymphalids. We calculated the D statistic for phylogenetic signal in a binary trait [73]. The value of the D statistic is based on the sum of changes between sister clades across the phylogeny. Highly clumped traits tend to have lower D values, closer to 0. In contrast more labile traits have higher values, with a D value of 1 representing a pattern close to phylogenetic randomness. We also compared the scaled value of the observed statistic to values generated under a model simulating under a Brownian model of phylogenetic structure and one resulting from no phylogenetic structure (each with 10,000 permutations) using the R package ‘Caper’ [74]. One can argue that a Brownian model of evolution is inappropriate for evaluating the phylogenetic signal of a non-evolved trait (albeit one correlated with evolved traits). Therefore we used a complimentary significance based approach to provide further support for these results, by testing for phylogenetic signal according to the mean phylogenetic distance (MPD) between extinct taxa. We used standardized effect sizes of MPD generated under null models of tip label randomization (999 runs) as implemented in the R package ‘Picante’ [75].

We tested for correlation between ecological and biogeographic traits of butterfly species and local extinction in BCI. Selection of these traits was guided by previous studies that considered the effect of anthropogenic disturbance on butterfly assemblages [2,6,10,11,76]. For this analysis, we considered only butterfly species that were common (> 10 individuals observed) during either the old or recent periods, or both (i.e. species in Categories 7 to 9). As the variables under investigation (see below) are likely to be phylogenetically conserved, we performed binary logistic regression in a phylogenetic context using the Phylogenetic Generalized Linear Models (PGLMs) of Ives and Garland [77] as implemented in the R package ‘phylolm’ [78]. By this approach we were able to assess the influence that each variable had on the probability of extinction independently of the phylogenetic relationships among butterfly species. The response variable was the probability of extinction, coded as either 0 (extinct species in Category 9) or 1 (common and settler species in Categories 7 and 8). The explanatory variables were host specificity (ordered categorical), host growth form (ordered categorical), geographic range (ordered categorical), color category (ordered categorical) and wing size (continuous; all variables are further described in S1 Text and S2 Fig). We used the “Logistic_MPLE” (Maximized Penalised Likelihood) method with a btol (bound on the linear predictor) of 1,000. Prior to analysis we tested for correlation among the explanatory variables by calculating the correlation co-efficients between each variable using a Spearmans rank correlation, none exceeded 0.57 (S1 Table). Furthermore, we also calculated the Generalized Variance Inflation Factors (VIFs) as implemented by M. Helmus (unpublished code available from GitHub as ‘AIC_func.r’) for our model without interaction terms, as none exceeded 1.6 we consider the inclusion of all explanatory variables to be valid. For our analysis we included all two way interactions apart from Size:Range, Growth Form:Color Category and Range:Color Category due to convergence problems caused by the inclusion of these terms (none of which were significant predictors when included in a separate model). Because no automated AIC based model simplification procedure is currently available for ‘phyloglm’ models we carried out backward step-wise model simplification based on the p-values of the model terms (terms with the largest insignificant p-values were removed first), with non-significant interaction terms being removed first. All models were then compared using the function ‘aictab.AICphyloglm’ written by M. Helmus and available (unpublished code available from GitHub as ‘AIC_func.r’). Because the flora of BCI is well-known [79] and has been censused regularly [80], we were able to assess whether the host plants of extinct butterfly species may still be present on BCI using recent plant records [81,82], as well as with expert botanist opinion (S. Aguilar, pers. comm.).

## Results

### Faunal composition and local species richness

The most species-rich butterfly families on BCI during the period 1923–2013 were Hesperiidae (33% of total number of species), Nymphalidae (31%), Lycaenidae (15%), Riodinidae (14%), Pieridae (4%) and Papilionidae (3%; full species list in S1 Appendix). The most species rich subfamilies were Pyrginae (including Eudaminae, to be consistent with Lamas, [42]), Theclinae, Hesperiinae and Riodininae, each contributing more than 80 species, whereas other butterfly subfamilies each contributed less than 40 species (S3 Fig). Most species (70%) in our list had valid BINs. Out of the total number of species in the list, 5% were cryptic species, 21% were species with sequences originating from elsewhere from BCI, and 45% were species with sequences originating from BCI (S3 Fig, S1 Appendix). For the old period, 64% of species had BINs whereas this proportion amounted to 90% for species collected during the recent period (S1 Appendix).

**Fig 1:**
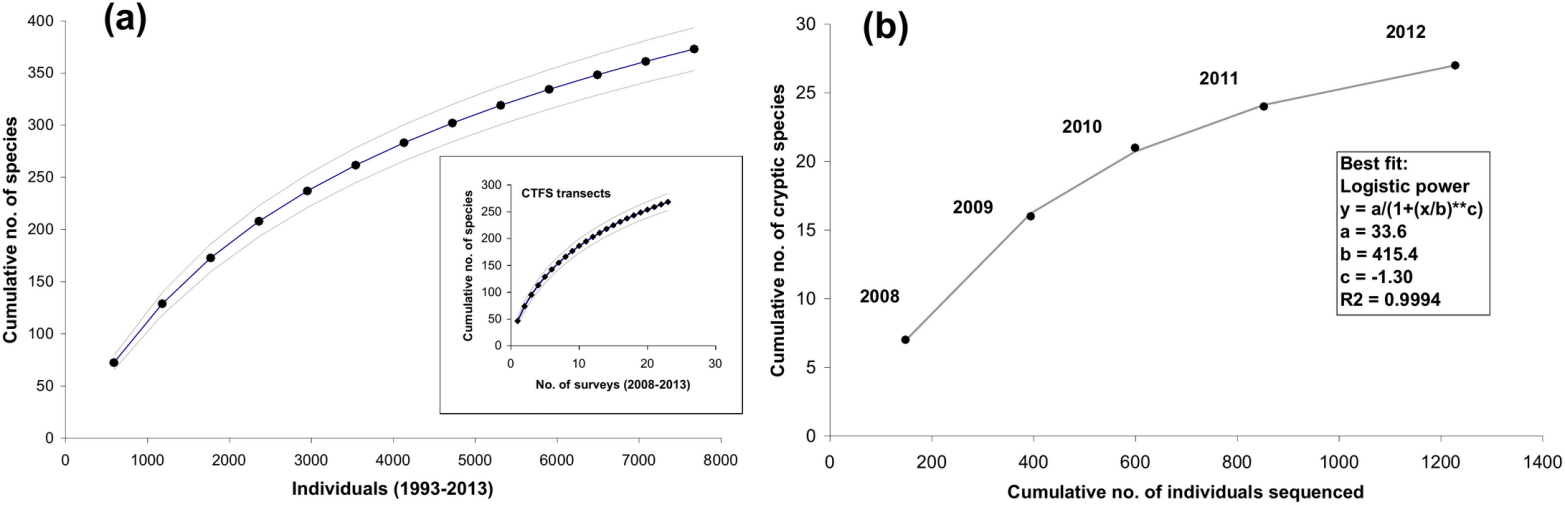
(a) Cumulative the number of individuals collected/observed plotted against the mean cumulative number of species collected/observed, for the recent period (1993–2013). Inset: cumulative no. of CTFS transects performed in the shady understory of BCI (2008–2013) plotted against the mean cumulative number of species collected/observed. Broken lines are 95% C.L. (b) Cumulative no. of individuals sequenced plotted against the cumulative no. of cryptic species discovered, for years 2008–2012. The grey line represents the best fit model, with its equation in inset.

Huntington [12] listed 267 butterfly species for BCI. Our full list for the 1923–2013 period includes 601 species. However, only 373 species (including cryptic species) were collected during the recent period (1993–2013) and the Incidence Coverage-based Estimator suggests that at least 514 ± 26.6 species (ICE ± SD) could have been collected on the island during recent times (Fig 1A). In the shady understory of BCI forests, using the CTFS transects, we collected 268 butterfly species during 2008–2013 (inset of Fig 1A). The ICE calculated with these data suggests that at least 390 ± 9.19 (SD) species may have been present in these forests during this period. Twenty-seven cryptic species were discovered after sequencing 1,228 individuals. The best fit model between the cumulative number of individuals and that of cryptic species suggests that a total of 34 cryptic species remain to be discovered on BCI (parameter *a* of the logistic power regression, Fig 1B). That number represents about 9% of the total number of species collected during the recent period. Further, theoretical taxonomic knowledge of BCI butterflies (S4 Fig) suggests that at the time of Linnaeus [83] only 4% of the species ever recorded on BCI had been formally described, whereas this proportion had already reached 91% at the time of publication of Huntington’s list [12].

### Comparison of old vs. recent periods

**Fig 2:**
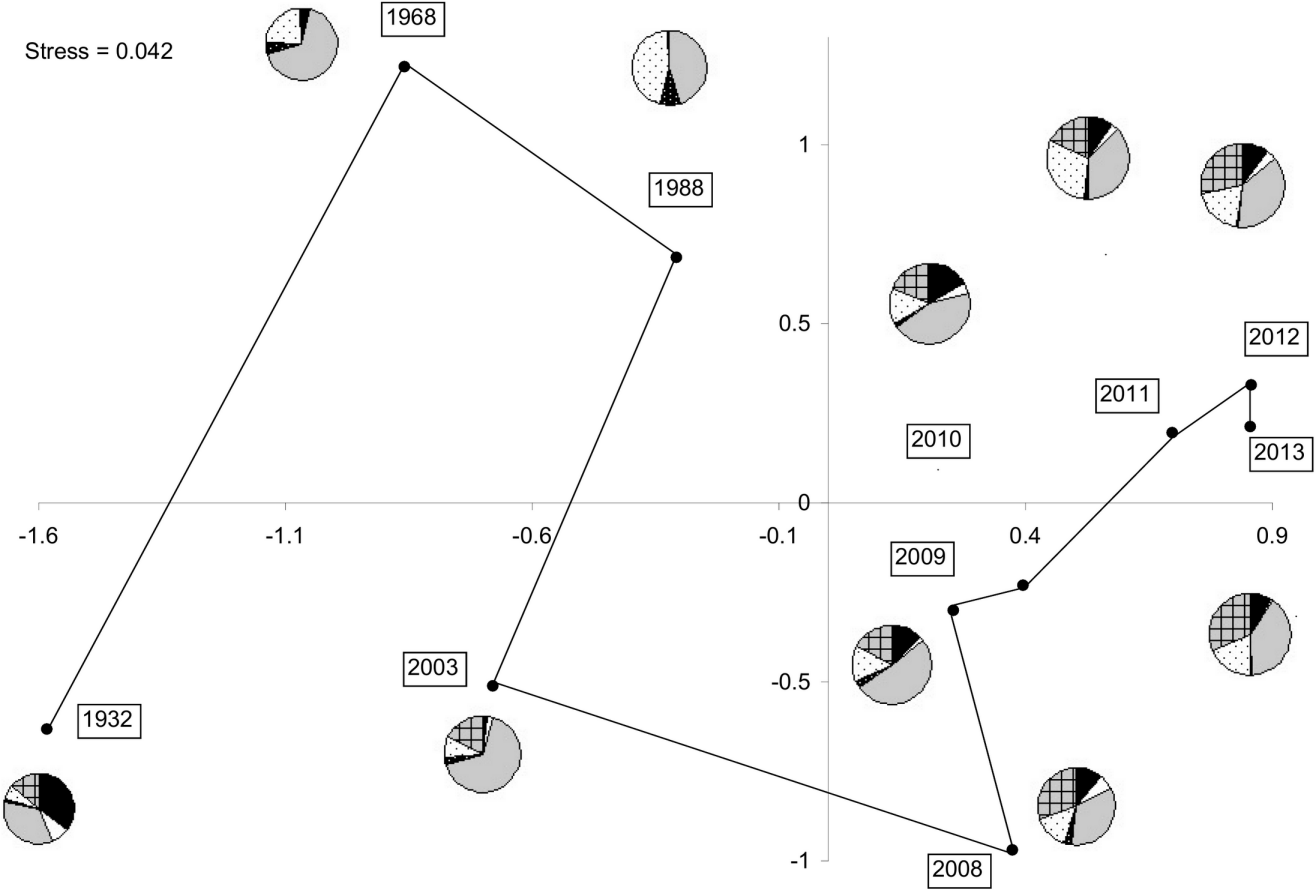
Plot of the scores of sampling years in axes 1 and 2 of the NMDS. Years are linked chronologically by a solid line. Pie charts indicate for each year the proportion of abundance accounted by (in clockwise order) Hesperiidae (black), Lycaenidae (white), Nymphalidae (grey), Papilionidae (black stippled), Pieridae (white stippled) and Riodinidae (grey squared).

The NDMS analysis clearly separated recent monitoring years (2008–2013) from older years, including 1932, along Axis 1 of the ordination (Fig 2). Multiple regressions indicated that only the variable Year itself explained significantly the scores of years on Axis 1 (F = 32.02, p<0.001, R^2^ = 0.775, n = 10), whereas sampling effort (no. of individuals recorded) explained to a lesser extent the formation of Axis 2 (F = 5.61, p = 0.045, R^2^ = 0.339, n = 10). The proportion of individuals recorded per family also varied along years, with, for example, a higher proportion of Pieridae (especially *Itaballia* spp.) and Riodinidae (*Detritivora* spp.) and a lower proportion of Hesperiidae being recorded during more recent years, as compared with Year 1932 (Fig 2). This was confirmed by a Chi-square test comparing the number of individuals recorded in each family for the old vs. recent period (Chi-square = 797.3, p < 0.0001, d.f. = 5). The Hesperiidae decreased from 36% to 10% of records, while Pieridae and Riodinidae rose from 6% to 20% and from 12% to 24%, respectively. In contrast, the number of species per family remained similar between the old vs. recent period (Chi-square = 5.3, p = 0.374, d.f. = 5). There was also a negative correlation between the number of individuals per species recorded in years 1932 and 2013 (r_s_ = -0.301, p<0.001, n = 327) and, similarly, between the number of individuals per species recorded during the old and recent periods (r_s_ = -0.252, p<0.001, n = 529).

### Locally extinct butterfly species

**Fig 3:**
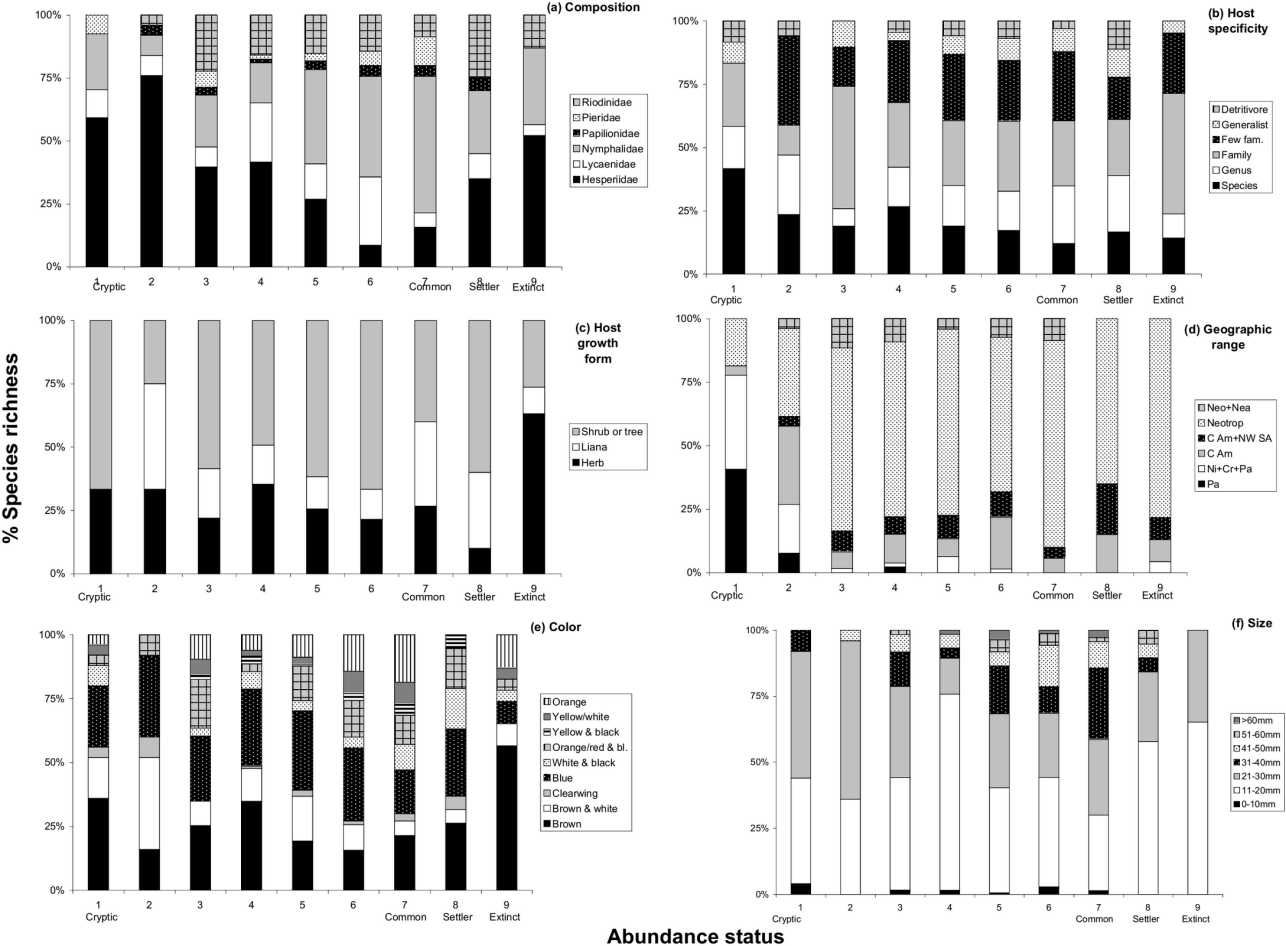
Details of the % distribution of species richness within the 9 categories of abundance status (Table 2) ordered by (a) faunal composition by families; (b) indices of host specificity; (c) host growth form; (d) indices of geographic distribution; (e) wing color pattern; and (f) wing size. For definition of (b), (d) and (e) indices, see S1 Text.

Cryptic and recently described species represented 5% and 4% of the 601 species collected during the period 1923–2013 (Table 2). Common and settler species represented 12% and 3% of the total, while locally extinct species only 4% (23 species). The vast majority of species (73%) had an unclear status of abundance according to our classification. Hence, of the species that had a higher abundance during 1923–2013 (Categories 7, 8 and 9 in Table 2), locally extinct species represented a much higher proportion, 20%. Thus, our analyses contrasted these three categories and, particularly, differences between common and extinct species. The distribution of butterfly families, of butterfly host specificity, host growth form, geographic distribution, wing color and wing size across the different categories of abundance status (as defined in Table 2) can be appreciated in Fig 3.

**Fig 4:**
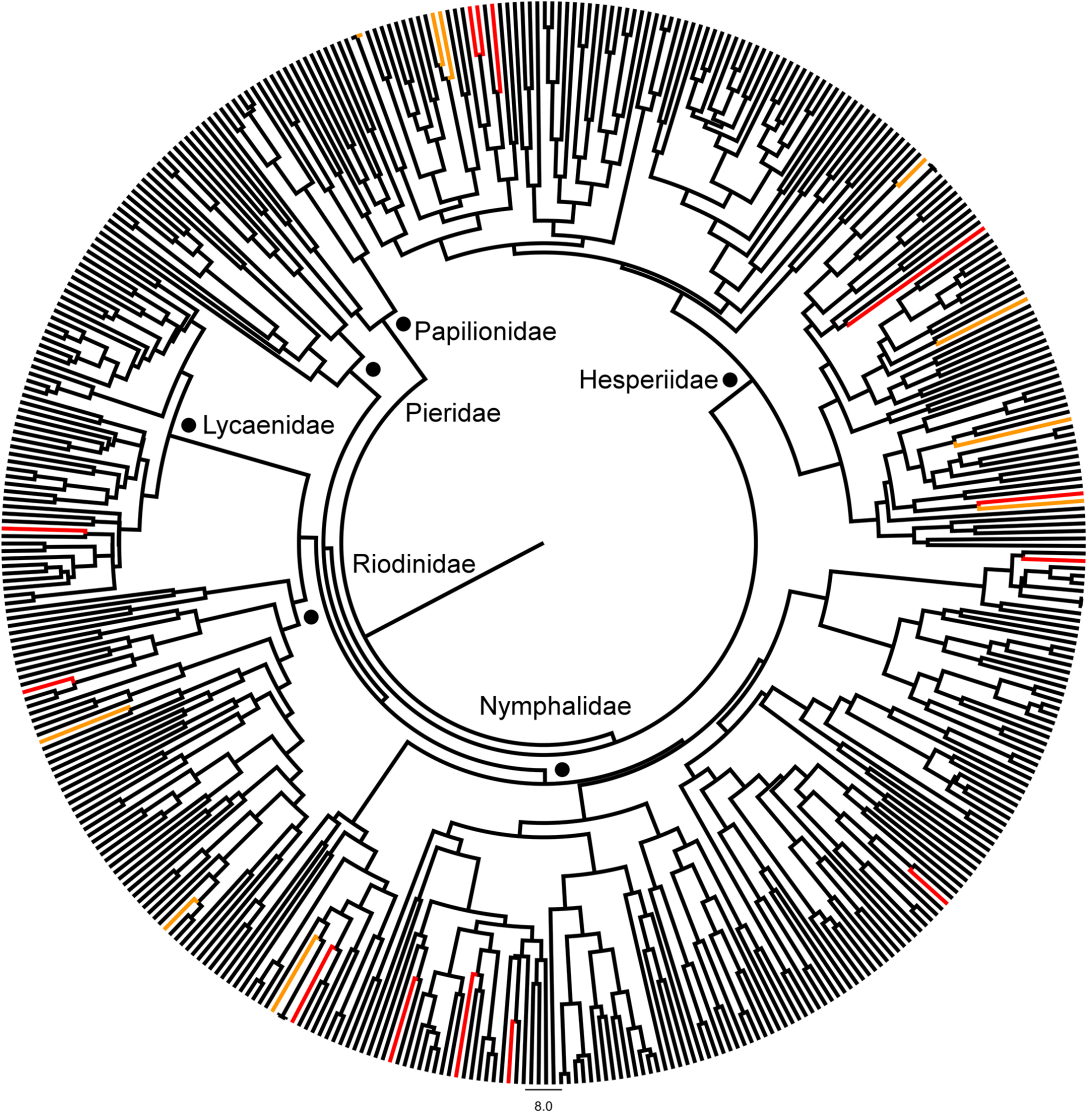
A maximum clade credibility consensus tree depicting the phylogenetic relationships between 451 butterfly taxa from six families (see text for details). Taxa marked in red (actual BIN used) or orange (replacement congeneric BIN used) represent taxa that were abundant in the 1923–1943 surveys but that were not found in the 1993–2013 surveys. Scale bar in millions of years.

Fig 4 provides a visual interpretation of how the extinct species clustered across the whole phylogenetic tree of BCI butterflies. Extinction status showed intermediate levels of phylogenetic signal when considering the whole butterfly phylogeny (D = 0.71, p (D > 0) = 0.009, p (D < 1) = 0.026) (S5 Fig). Extinction status showed reasonably strong phylogenetic signal when considering only the family Hesperiidae (D = 0.13, p (D > 0) = 0.428, p (D < 1) = 0.003) (S6 Fig). Nymphalidae showed no significant clumping across the phylogeny (D = 1.21, p (D > 0) = 0.006, p (D < 1) = 0.781) (S7 Fig). Significance tests of phylogenetic signal according to MPD broadly agreed with our results based on D. There was significant clumping of extinction status across the whole butterfly phylogeny (MPD observed = 133.339, MPD random mean = 136.985, p = 0.022) and across the hesperiid phylogeny (MPD observed = 106.975, MPD random mean = 111.301, p = 0.042) but no significant clumping across the nymphalid phylogeny (MPD observed = 121.045, MPD random mean = 123.576, p = 0.236).

The simplest model with no non-significant terms was selected using differences in AICc scores. In this model growth form (z = 3.083, p = 0.002) and wing size (z = 2.391, p = 0.017) had a significant effect on the probability of extinction, with smaller butterflies feeding on herbaceous plants more likely to go extinct. Host specificity was also retained as a significant term (z = -2.556, p = 0.011). The significant interaction terms Growth Form:Size (z = -2.154, p = 0.031) and Host Specificity:Range (z = 2.150, p = 0.032) were also retained in the model. The p-values derived for each coefficient are Wald type p-values conditional on the phylogenetic correlation parameter (alpha), which was approximately 0.0132 for each model.

The 23 species listed as extinct are further discussed, in terms of possible misidentifications and loss of host-plant species, in S2 Appendix. This list includes 12 Hesperiidae, 7 Nymphalidae, 3 Riodinidae and 1 Lycaenidae. In 13 cases, their host plants included herbs, in 6 cases either palms, shrubs or trees, in two cases lianas, and in two cases were unknown. The most common case included hesperiids feeding on herbs (8 cases). In contrast, Category 7 (common species) included 38 Nymphalidae, 11 Hesperiidae, 8 Pieridae, 6 Riodinidae, 4 Lycaenidae and 3 Papilionidae. For 19 of these 70 species, their host was either a tree or a shrub. The most common case in Category 7 included a nymphalid feeding on a liana. For 17 locally extinct species, we provide evidence that their host plant(s) are still extant on BCI and that extinction is unlikely to result from a decrease in host plant(s) abundance. However, local extinction of host plants may have resulted in the extinction of four butterfly species on BCI (S2 Appendix). These include *Corticea noctis* feeding on sugarcane, *Staphylus mazans* and *S*. *musculus* feeding on Amaranthaceae and Chenopodiaceae (but these two species are likely to be misidentified see S2 Appendix), and *Ithomia iphianassa* feeding on *Capsicum* and *Witheringia*. Sugarcane and *Capsicum* were cultivated on BCI during the old period.

## Discussion

### Size of the butterfly species pool on BCI

Some 601 butterfly species have been recorded during 1923–2013 on the 1,542 ha Barro Colorado Island. This number is plausible given that in Costa Rica, a complex mosaic of 120,000 ha of dry, cloud and rain forest over 0–2,000 m elevation hosts more than 978 butterfly species [84], suggesting that about 40% of all butterfly species estimated for Panama might occur on BCI ([85]: 1,550 species). Unlike Janzen *et al*. [84] we could not systematically sequence each butterfly specimen collected during the recent period. Thus, our total estimate of 34 cryptic species on BCI (9%, against 16% for Janzen *et al*. [84]) probably represents an underestimate. These cryptic species were not included in the old vs. recent period comparisons. They could potentially complicate these comparisons, because of possible misidentifications (S2 Appendix). The total number of species collected on BCI may be also inflated by misidentifications but we have tried, whenever possible, to assist our identifications with DNA barcoding.

During the recent period 373 species were collected on BCI and the ICE estimator suggests that at least 514 species could actually be present there. However, that does not imply that all of these species are resident (i.e., breeding) on the island. Extended flights over water near BCI are a likely occurrence for many butterflies [27]. They may originate from the nearest forests and open areas close to BCI and cross the water channel that is 0.5–3.5km wide, depending on the location. Good dispersers may not necessarily be ovipositing on the island. Poor dispersers (smaller species) may not be able to cross the water channel. Dynamics of colonization-extinction for BCI butterflies would warrant further studies.

The steep rise in the accumulation of butterfly species in CTFS transects performed in the shady understory suggests a large species pool and, possibly, dispersal in the forest of species that usually prefer to fly in the canopy or open habitats. Because BCI is currently ca. 100% covered by forest, CTFS transects are probably representative to estimate the number of resident (breeding) species on BCI, which we estimated to be 390 species. We also note that 36% of the 70 common species (Category 7) have been reared from host-plants on BCI and thus are more likely to be breeding on the island (datasets Aiello and Coley, Table 1).

### Challenges in ascertaining local extinction rates

When Huntington published his checklist [12], taxonomical knowledge of BCI butterflies was good, with 91% of today’s fauna already known. Therefore it is sound to compare his checklist with butterfly collections described after 1943, excluding cryptic species, which represent 5% of the species ever collected on the island. Taxonomic knowledge of BCI butterflies mirrored advances in Neotropical lepidopterology, with early descriptions (Linnaeus, Fabricius), followed by the monumental works of Bates [86], Godman and Salvin [87] and the recent surge of cryptic species discovered by molecular techniques [84].

Many species in our classification had an unclear status of abundance (Categories 3 to 6, 63% of the total number of species in the list), because of the long tail of species-abundance distributions typical of tropical forest habitats [88]. This presents a common challenge to studies of tropical insect conservation. We need to discuss the cases where there is reasonable evidence for local extinction, i.e. species that were common during the old period but have not been collected recently despite a much higher sampling effort. Though it may yield extremely conservative estimates of local extinction we must ignore species considered rare during the old period (Category 4, 132 species) because it is very difficult to ascertain whether they may be extinct on BCI today.

Misidentifications of extinct species cannot be discounted, but they should at most include 9 out of the 23 species listed in our extinct category. Eleven of the 23 extinct species have BINs (48% of species in Category 9; S2 Appendix), and despite high sampling efforts these BINs have not been sequenced from specimens collected recently on BCI. Still, our extinct species category includes many taxonomically-challenging taxa, for which we cannot exclude misidentification (11 out of 23 species, 48%). Ten of the 12 hesperiids listed as extinct were identified by Ernest L. Bell, who was one of the foremost authorities on New World hesperiids. Very few of his 200+ species and subspecies have fallen into synonymy [89], making it unlikely that he misidentified the hesperiids in our extinct species list. However, there are possible misidentifications, such as the two *Staphylus* spp., which do not occur in Panama (A.D. Warren, pers. obs.). Huntington’s specimens [12] were identified by himself, assisted by Bell, Curran and Watson. Sheldon’s [45] specimens were identified by Arthur Hall. The highest probability for misidentification concerns three extinct species uniquely recorded by Sheldon [45]: *Pythonides herennius*, *Ithomia iphianassa* and *Emesis cerea*, but that cannot be confirmed without vouchers. Finally, misidentifications are also possible among recent specimens, which could belong to species listed as extinct. That would be most likely for the four indistinct species without BINs: *Corticea noctis*, *Pareuptychia ocirrhoe* and the two *Staphylus* mentioned above.

### Local butterfly extinction and relationships with host plants

What proportion of the probable pool of ca 390 resident and extant species can we consider as locally extinct on BCI? The short answer to this is 23 spp. / (390 + 23 spp.) or 6% if we include all extinct species in Category 9. More conservatively we could include only the 12 species in Category 9 for which the probability of misidentification is very low, decreasing the proportion of extinct species to 3%. A more radical approach would consider all 132 species in Category 4 as being extinct or in danger of extinction, raising the proportion of extinct species to 38%. Therefore, extinction rates may range between 3% and 38% of resident species, with a most probable estimate of 6%, as our data and analyses suggest. Besides, many of the species locally extinct on BCI have been collected recently in the “Canal Area” adjacent to BCI (S2 Appendix).

Phylogenetic signals of extinction were of intermediate strength when they were present, and occurred on distant phylogenetic branches within several families rather than in distinct clades. The most speciose family, the Hesperiidae, showed the largest phylogenetic signal as each subfamily had a clade with a higher extinction risk. In contrast the second most speciose family, Nymphalidae, showed no phylogenetic signal with respect to extinction risk (if anything borderline over dispersion was detected). These results may give some small insight into how host use may play a role in species extinctions (in addition to the results of our logistic regression). Many members of the family Hesperiidae feed on herbs and palms, whilst members of Nymphalidae have a much wider range of host plants [72]. The more clustered extinctions in Hesperiidae may reflect fine scale host conservatism within the family, whilst it is possible that nymphalid species may be pre-adapted to host plant loss. The traits most likely to influence the probability of extinction were host plant growth form, butterfly wing size and host specificity (specialist vs. generalist species), independent of the phylogenetic relationships among butterfly species. Size of geographical range and wing color patterns (dull vs. brightly colored species as a proxy for bias in species detection) had no significant influence on the probability of extinction. Our most likely candidates for extinction were small hesperiids feeding on herbs (35% of extinct species). However, contrary to our working hypothesis, extinction of these species cannot be imputed to loss of host plants on BCI. In most cases, those host plants remain extant on BCI, although their recent abundances have not been ascertained. The most drastic and recent changes in BCI vegetation have been the closure of forests and loss of open habitats. In 1929, herbs represented 48% of plant species on BCI [39]. Recent figures are not available because CTFS is monitoring trees and lianas on the island, not herbs [38]. However, many open habitats have been lost or fragmented, and many herb species must subsist now at lower and more fragmented densities than before. Many tropical butterfly species have been shown to have short dispersal distances (< 200m; [90]). The decline in herbaceous hosts on BCI may have reduced the fitness of smaller butterfly species (poor dispersers) feeding on herbs, possibly leading to local extinction. The significant interaction between host growth form and butterfly size in the phylogenetic generalized linear model describing the probability of extinction would lend credence to this interpretation. In temperate areas, some butterfly species occupy wide areas as “transient” colonies/populations that move around within that general range as habitats shift [91]. That scenario may apply also to some of our locally extinct species. Other factors, perhaps related to global climate change [92], possibly have pushed some BCI species towards extinction, although this is difficult to discuss without good baseline data and on-going monitoring programs.

## Conclusions and Implications

A small number of butterfly species (6% of the resident pool) have probably become locally extinct on BCI. The populations of these species probably declined as a result of the loss and fragmentation of habitats where their herb host was growing. A remedy to this situation, if one wants to re-install these butterfly species on BCI, would be to manage the forest and allow locally the herbaceous vegetation to grow back. This is common practice to conserve butterflies of dry meadows in Europe [93]. Small amounts of disturbance are beneficial to conserve a high proportion of biodiversity [94]. However, even if host plants are present, local conditions may not be suitable for butterflies to complete their life cycles on them, as is probably true for most of the extinct species reported in this study. Knowledge of species’ life history appears paramount to conserve them, even for tropical invertebrates.

Karr [34] reported that 50–60 species of birds were lost to BCI since its isolation from the mainland, as a result of several factors. In comparison, far fewer butterfly species have been lost from BCI in recent times. Butterflies are known to be able to maintain viable populations in tiny habitat fragments of a few to hundred hectares [9]. Our data confirm that small preserves may be far more effective to conserve invertebrates than vertebrates and, therefore, should not necessarily be neglected from a conservation viewpoint.

Butterflies are popular indicators to monitor forest disturbance, mainly because of logistical and visibility advantages over other insect taxa [95]. Changes in the absence and presence of species over years were difficult to evidence in our study, as indicated by the high numbers of species in Categories 3 to 6. Changes in butterfly composition over the years on BCI were more clearly evidenced by the abundance of a few species limited to the forest interior (Y. Basset *et al*., unpubl. data; *Itaballia* spp.; Fig 2). Thus, for long-term monitoring of butterflies in tropical rainforests, analyses of population changes of particular species may be more informative than synecological analyses of whole assemblages based on presence-absence data.

Lastly, irrespective of their conversion into plantations and secondary logged forests [96], tropical rainforests are going to lose butterfly and insect species in the relatively short-term by either natural succession or through the effects of global climate change. In this regard the 155 butterfly species listed in our Categories 4 and 9 probably represent potential candidates for extinction, but there is great uncertainty about their abundance status. To refine recent extinction rates of insects in tropical rainforests, we imperatively need to invest more resource in detailed long-term insect monitoring with adequate frequency.

## Supporting Information

S1 Appendix List of butterfly species (Hesperiidae & Papilionoidea) collected or observed on Barro Colorado Island, for the period 1923–2013. The number of specimens is summed for the old (1923–1943) and recent (1993–2013) periods, as well as for the whole study period (1923–2013, Abundance), and detailed for each year on record. When available, Barcode Index Numbers (BINs) are indicated for each species. Indices of host specificity, geographic range and abundance status are also listed for each species, as well as congeneric replacements for extinct species, for which barcodes were unavailable (see text). A few isolated records were also extracted from the specialised literature and those are listed in the last column.
(XLS)

S2 Appendix Commented list and salient characteristics of butterfly species in the "extinct" category. Abundance refers to total records 1923–2013. “1923–1943” and “1993–2013” to the number of records during the old and recent periods, respectively.
(XLS)

S1 Fig Records of butterfly collected or observed on BCI, for the period 1923 to 2013 (log scale). (TIF)

S2 Fig Examples of wing color patterns: Brown (1–5), Brown & white (6–10), Clearwing (11–13), Blue (14–18), White & black (19–23), Orange/red & black (24–29), Yellow & black (30–32), Yellow/white (33–35), Orange (36–42). (TIF)

S3 Fig Details, for the main butterfly subfamilies, of the number of species without Barcode Index Number (BIN), of species with BIN originating from specimens not collected on BCI, of species with BIN originationg from specimens collected on BCI and of species not formally described but with distinct BIN ("cryptic"). (TIF)

S4 Fig Theoretical taxonomic knowledge of BCI butterflies relative to present days. Plot of the cumulative number of species described (or observed in the case of cryptic species) against the year of description. Text boxes indicate landmarks in taxonomic knowledge and the % of species known at that time relative to the total number of species presently known (600 spp.).
(TIF)

S5 Fig A plot of D values vs. density under two null models. The red density plot is generated under random phylogenetic signal and the blue density plot is generated under Brownian motion. The black bar (D = 0.71) represents the observed D value for extinct species across the wider butterfly phylogeny. This value does not differ from expectations under a random phylogenetic model.
(TIF)

S6 Fig A plot of D values vs. density under two null models. The red density plot is generated under random phylogenetic signal and the blue density plot is generated under Brownian motion. The black bar (D = 0.13) represents the observed D value for extinct species across the phylogeny of the family Hesperiidae. This value is has a higher probability of coming from a distribution generated under Brownian motion than a distribution generated under a random phylogenetic model.
(TIF)

S7 Fig A plot of D values vs. density under two null models. The red density plot is generated under random phylogenetic signal and the blue density plot is generated under Brownian motion. The black bar (D = 1.21) represents the observed D value for extinct species across the phylogeny of the family Nymphalidae. This value does not differ from expectations under a random phylogenetic model.
(TIF)

S1 Table Lower Spearman coefficient correlation matrix between independent variables included in the Phylogenetic Generalized Linear Model. (DOC)

S1 Text Supplementary methods. (DOC)
