# Supplementary figures and images for: The Butterflies of Barro Colorado Island, Panama: Local Extinction since the 1930s
Source: PLoS One. 2015 Aug 25;10(8):e0136623. doi: 10.1371/journal.pone.0136623 (PMC4549329; doi:10.1371/journal.pone.0136623)

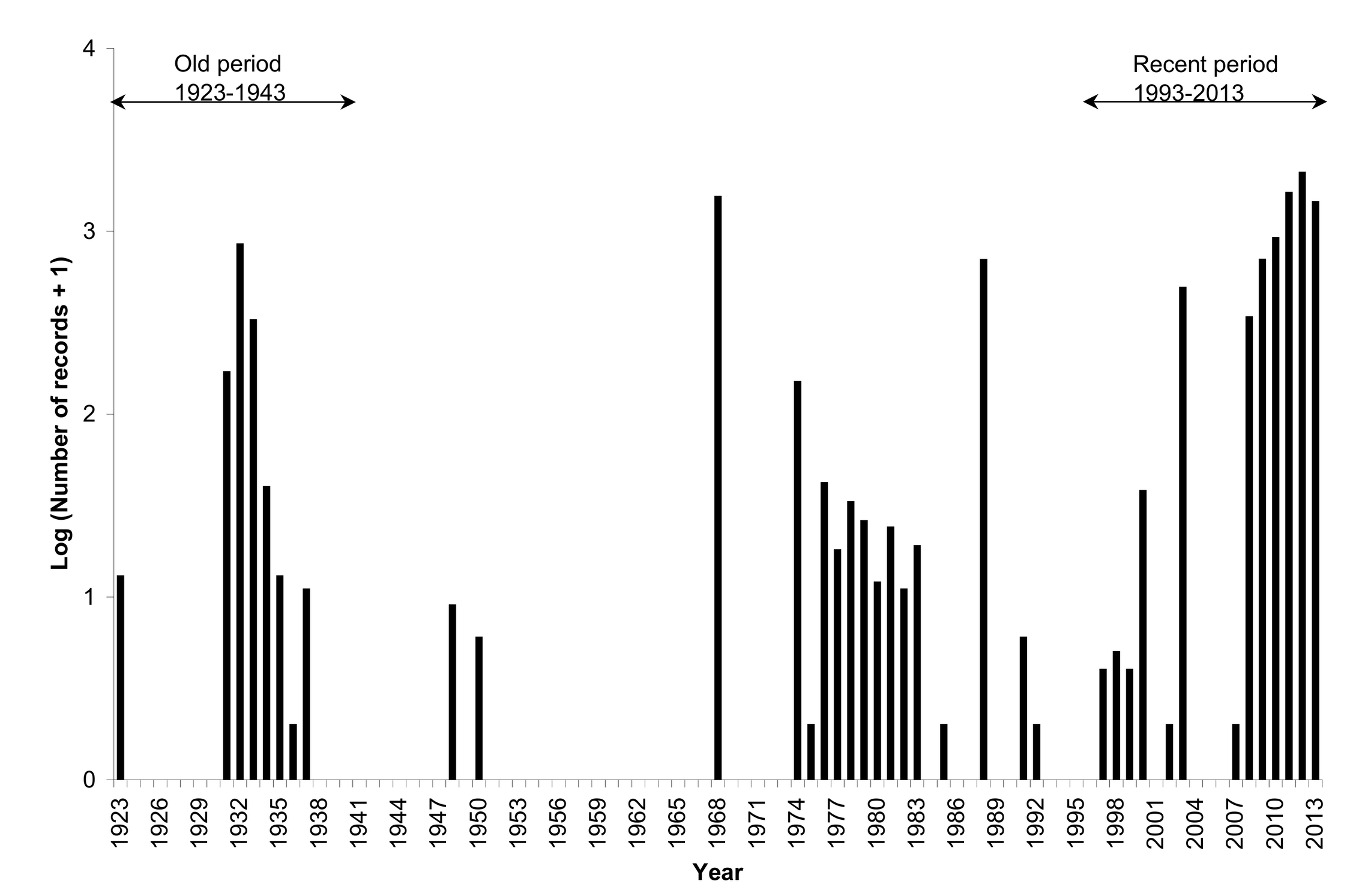

Supplement: S1 Fig — (TIF) [file pone.0136623.s003.tif]

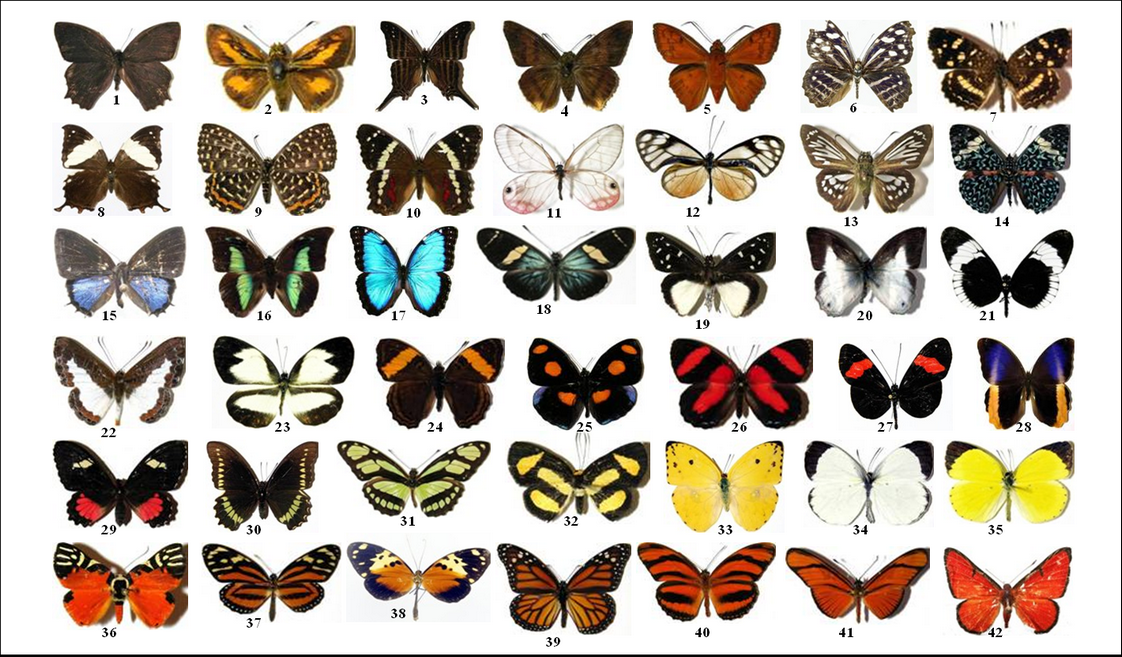

Supplement: S2 Fig — (TIF) [file pone.0136623.s004.tif]

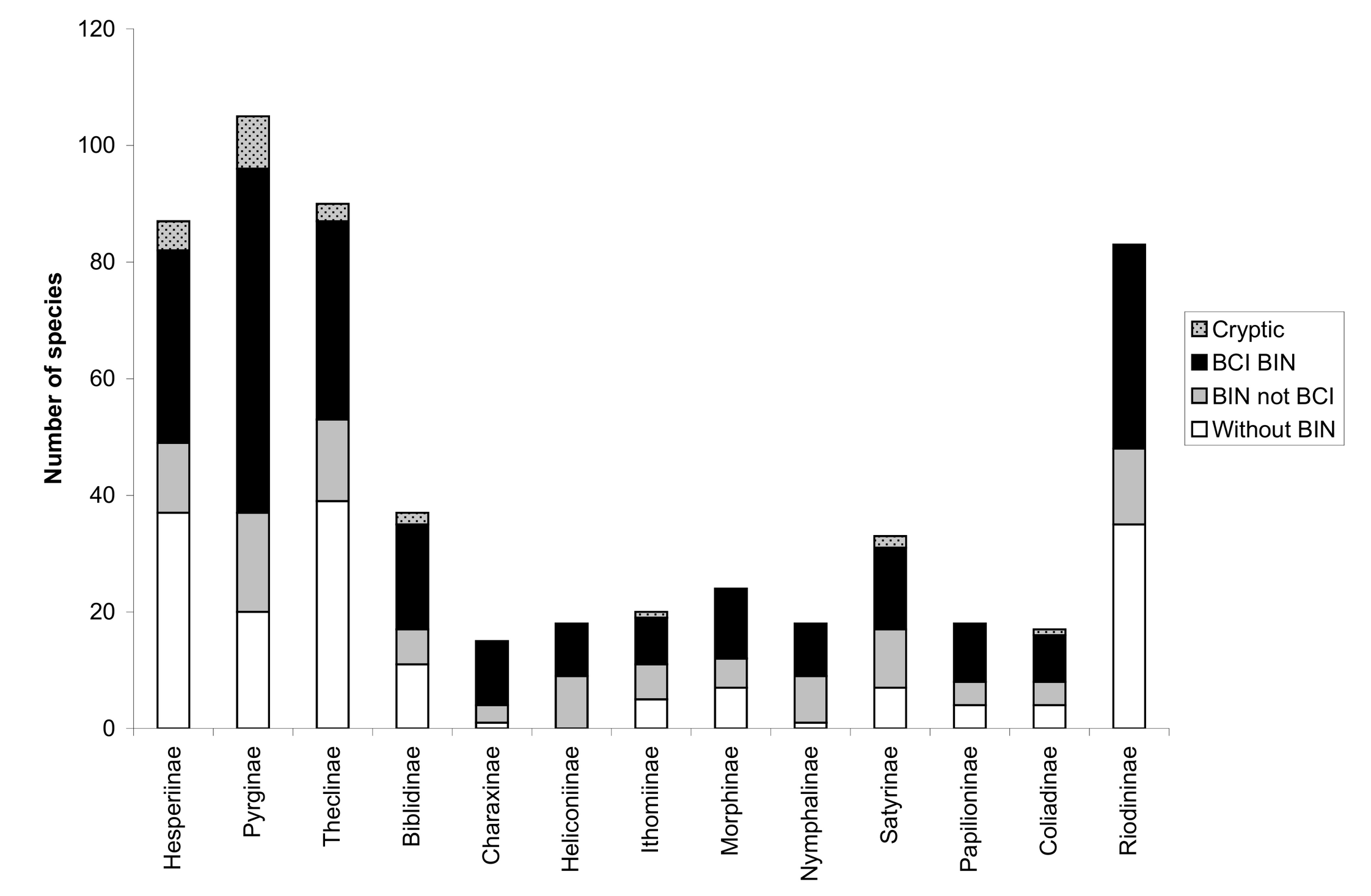

Supplement: S3 Fig — (TIF) [file pone.0136623.s005.tif]

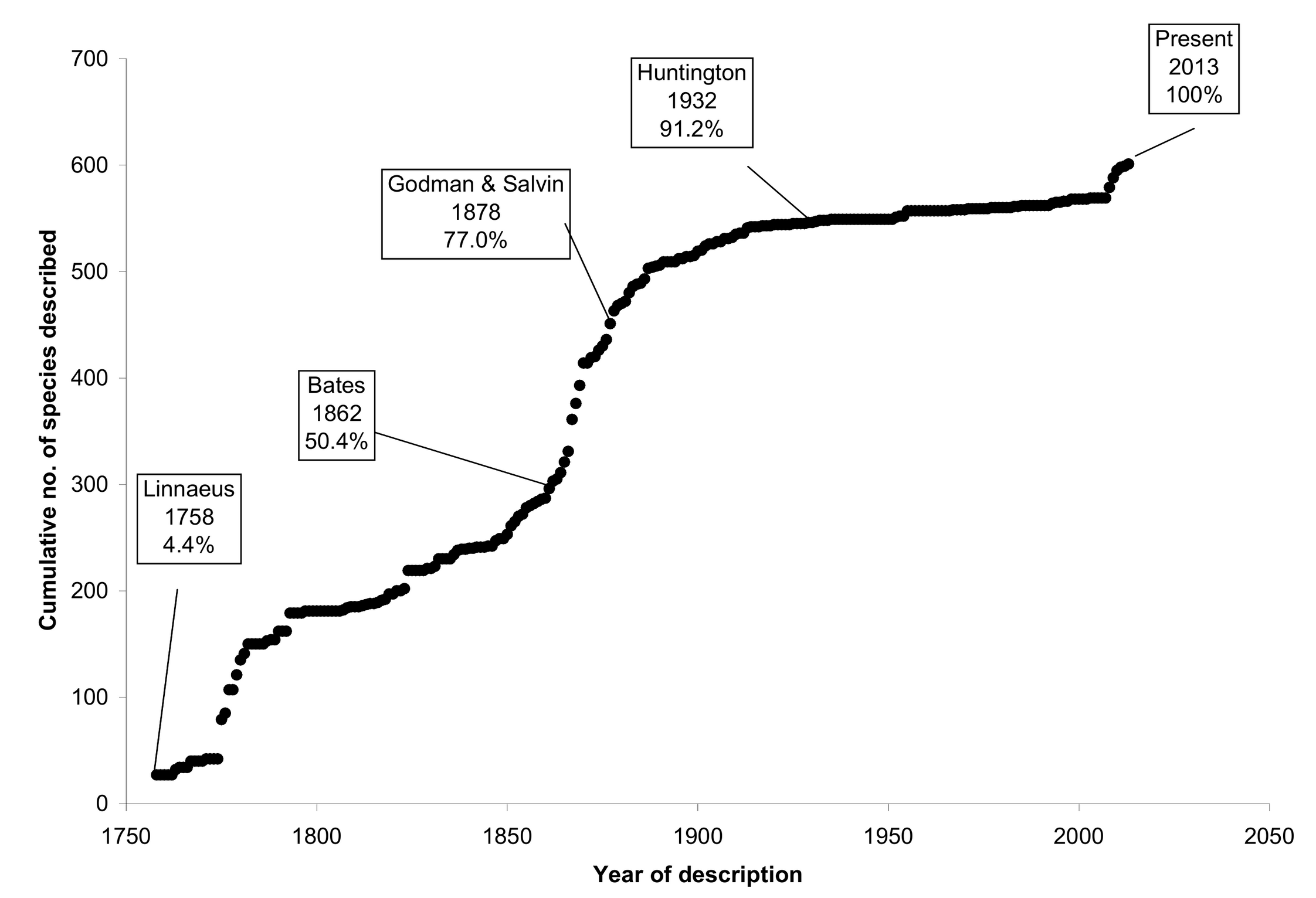

Supplement: S4 Fig — Plot of the cumulative number of species described (or observed in the case of cryptic species) against the year of description. Text boxes indicate landmarks in taxonomic knowledge and the % of species known at that time relative to the total number of species presently known (600 spp.). (TIF) [file pone.0136623.s006.tif]

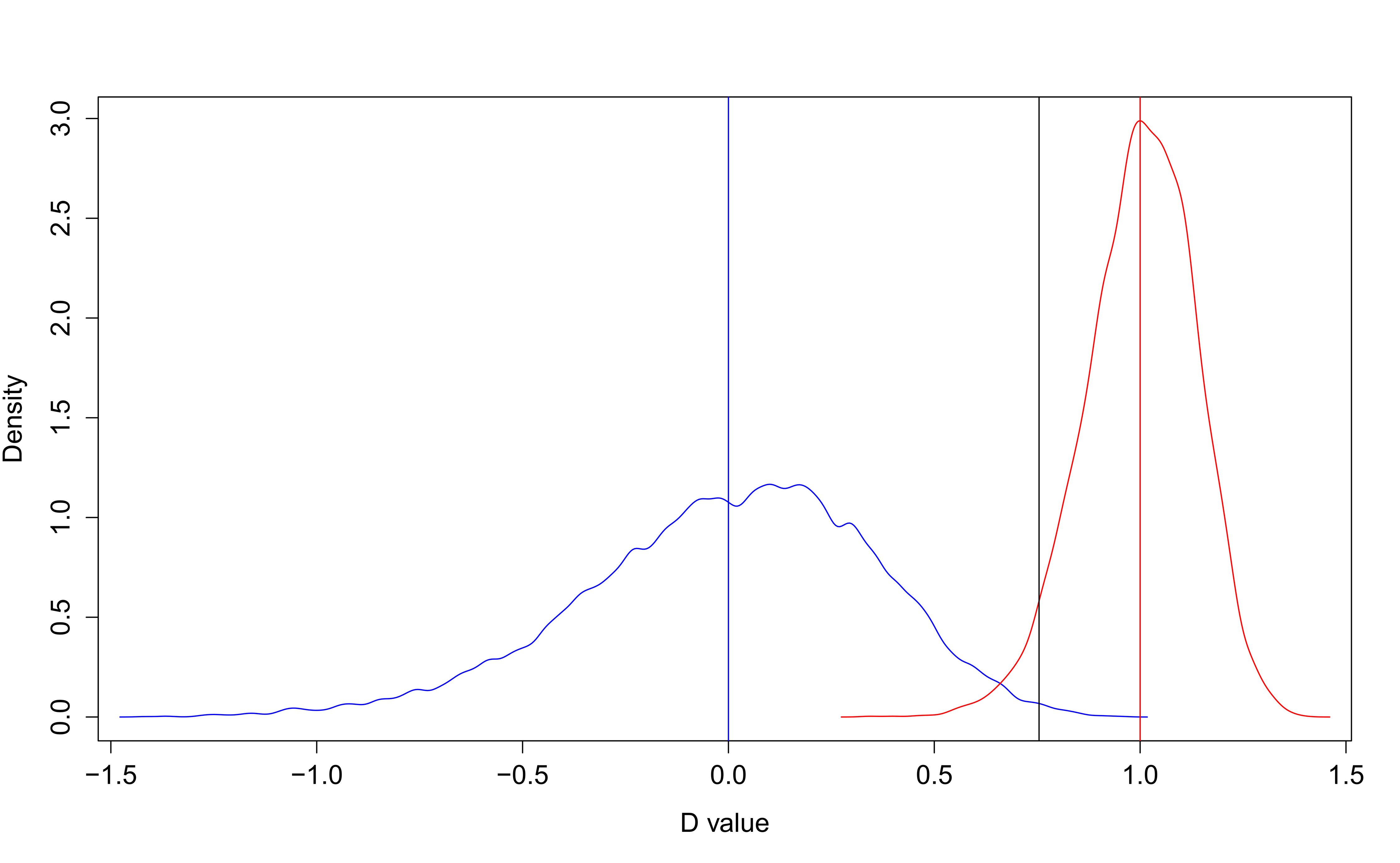

Supplement: S5 Fig — The red density plot is generated under random phylogenetic signal and the blue density plot is generated under Brownian motion. The black bar (D = 0.71) represents the observed D value for extinct species across the wider butterfly phylogeny. This value does not differ from expectations under a random phylogenetic model. (TIF) [file pone.0136623.s007.tif]

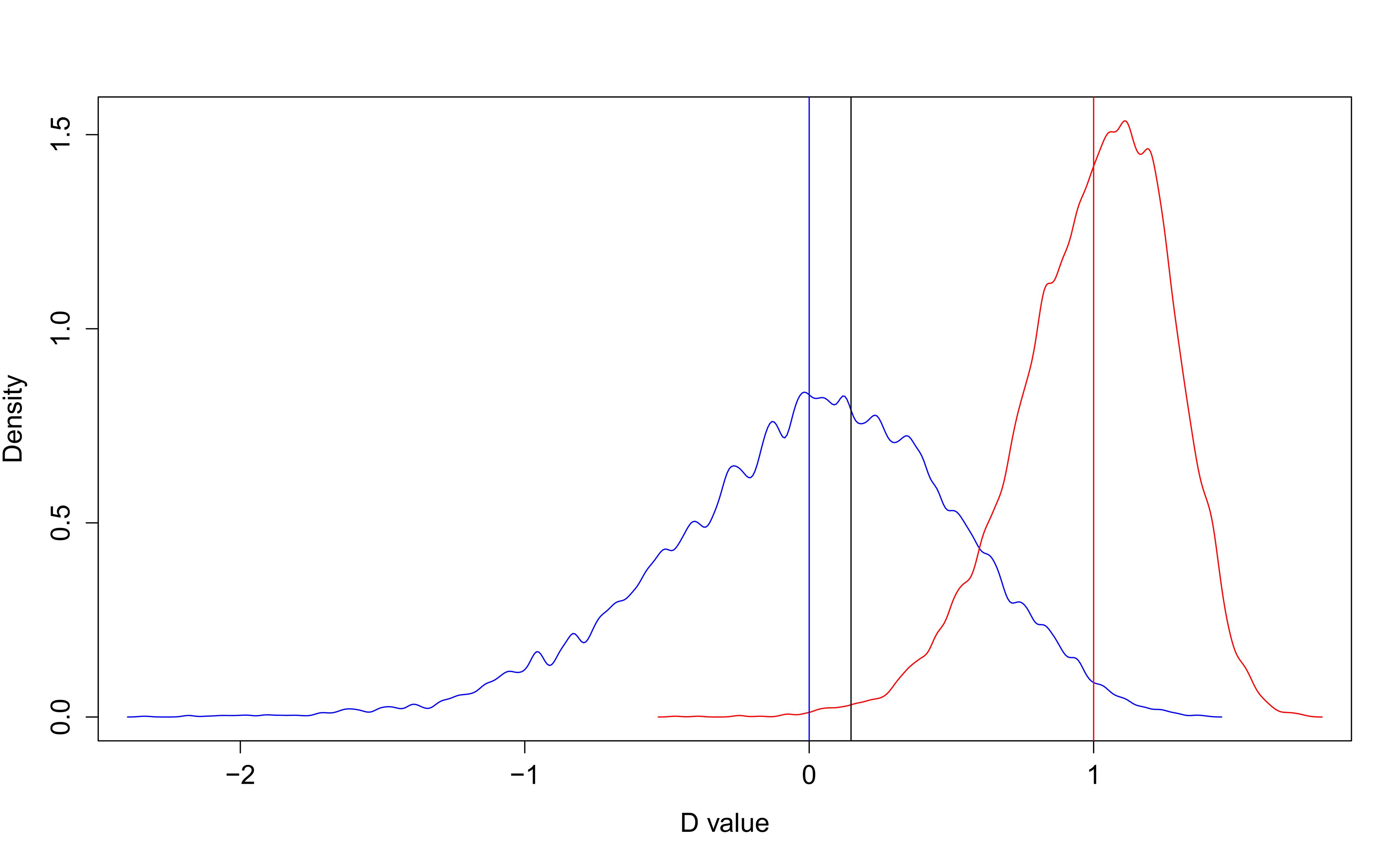

Supplement: S6 Fig — The red density plot is generated under random phylogenetic signal and the blue density plot is generated under Brownian motion. The black bar (D = 0.13) represents the observed D value for extinct species across the phylogeny of the family Hesperiidae. This value is has a higher probability of coming from a distribution generated under Brownian motion than a distribution generated under a random phylogenetic model. (TIF) [file pone.0136623.s008.tif]

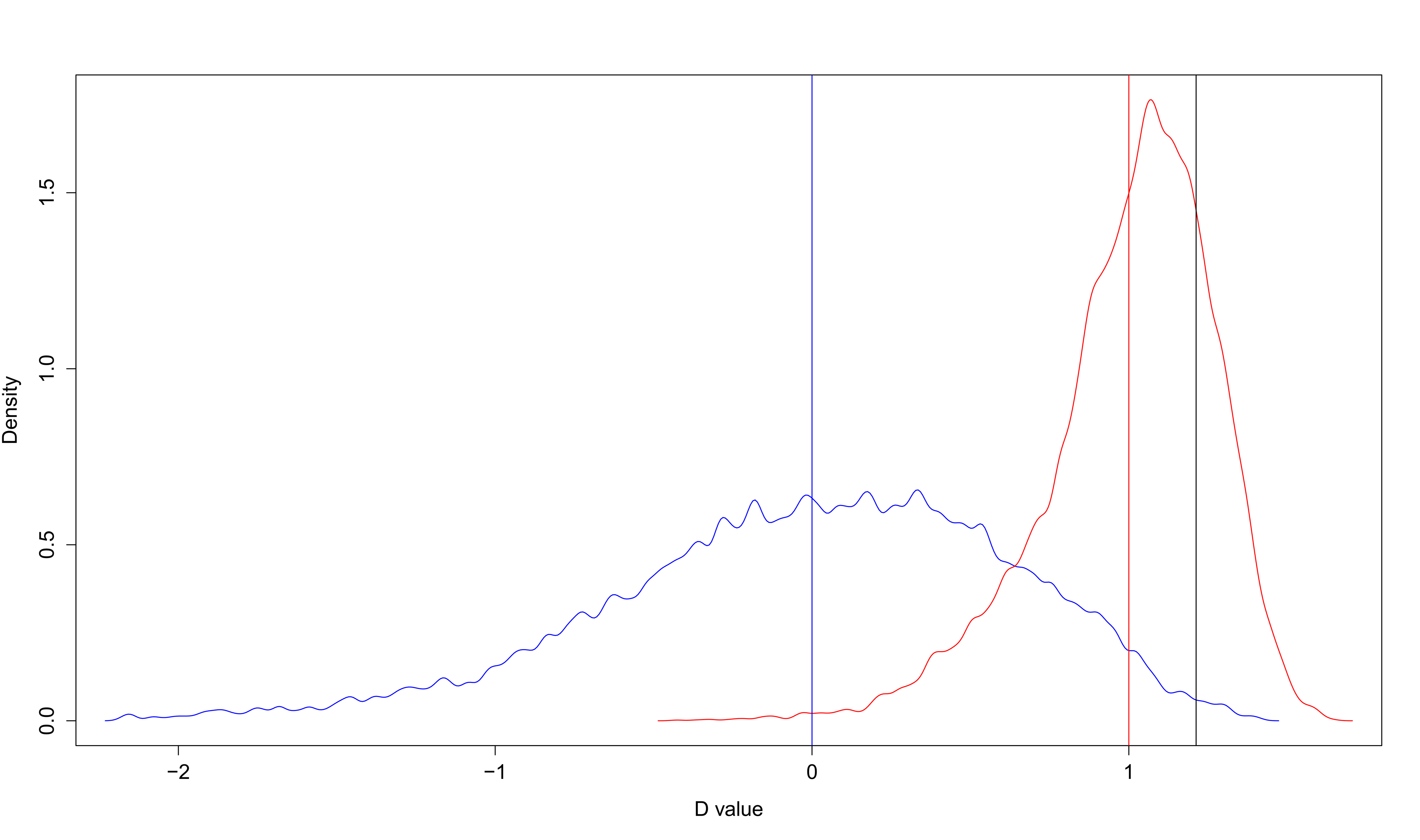

Supplement: S7 Fig — The red density plot is generated under random phylogenetic signal and the blue density plot is generated under Brownian motion. The black bar (D = 1.21) represents the observed D value for extinct species across the phylogeny of the family Nymphalidae. This value does not differ from expectations under a random phylogenetic model. (TIF) [file pone.0136623.s009.tif]
