# Supplementary material for: The Butterflies of Barro Colorado Island, Panama: Local Extinction since the 1930s
Source: PLoS One. 2015 Aug 25;10(8):e0136623. doi: 10.1371/journal.pone.0136623 (PMC4549329; doi:10.1371/journal.pone.0136623)
Supplement: S1 Table — (DOC) [file pone.0136623.s010.doc]

**Table S1.** Lower Spearman coefficient correlation matrix between independent variables included in the Phylogenetic Generalized Linear Model. Specificity = host specificity, Growth = host growth form, Range = geographic range, Color = category of wing color, Size = wing size.

|  | Specificity | Growth | Range | Color | Size |
| --- | --- | --- | --- | --- | --- |
| Specificity | 1.00 |  |  |  |  |
| Growth | 0.28 | 1.00 |  |  |  |
| Range | 0.05 | -0.02 | 1.00 |  |  |
| Color | 0.01 | 0.39 | 0.23 | 1.00 |  |
| Size | 0.03 | 0.17 | 0.29 | 0.57 | 1.00 |
