# Supplementary material for: The Butterflies of Barro Colorado Island, Panama: Local Extinction since the 1930s
Source: PLoS One. 2015 Aug 25;10(8):e0136623. doi: 10.1371/journal.pone.0136623 (PMC4549329; doi:10.1371/journal.pone.0136623)
Supplement: S1 Text — (DOC) [file pone.0136623.s011.doc]

### S1 Text. Supplementary Methods

All butterfly species recorded on BCI during 1923-2013 were characterized by the following suite of life-history traits and morphological characters, which were as far as possible ordered into gradients:

**1. Host specificity.** We compiled larval host plants from references indicated below [16, 49,50,97-113] and derived a simple index of host specificity as: 1 = restricted to one plant species; 2 = restricted to one plant genus; 3 = restricted to one plant family; 4 = restricted to a few plant families; 5 = wide generalist; 6 = detritivorous.

**2. Host growth form.** Coded for the analyses as 1 = herb (including bamboo and grasses); 2 = palm; 3= tree or shrub; 4 = liana; 5 = a combination of previous categories; 6= dead matter.

**3. Geographic range.** We modified as follows the system of Thomas [2] to summarize the geographical distribution of our BCI species: 1 = restricted to Panama; 2 = Nicaragua, Costa Rica and Panama; 3 = (i) Central America (to Panama) or (ii) Nicaragua to NW South America; 4 = Central America to NW South America; 5 = Widespread in Central and South America (Neotropical); 6 = Neotropical and part of Neartic. Geographic range was determined using the information provided by Savela [110].

**4. Wing color patterns.** We used a modified system from Burd [114] to assess possible biases in human observers and/or emphasize different challenges in identifying visually species among sites. We use this system as a proxy for species detectability. We do not use it to discuss the ecological significance of butterfly color patterns. The nine categories of wing color are ordered from the most difficult to the easiest species to detect in the understory of a tropical rainforest.

1. *Brown*: wing area almost entirely blackish, dark brown, light brown or gray; or a combination of these colors.

2. *Brown & white*: wing area blackish, dark brown, light brown or gray with patterns of points, lines or narrow bands white or very pale yellow; these patterns representing < 20% of the total wing area.

3. *Clearwing*: 50% or more of the area without scales.

4. *Blue*: dark wing area with patterns of points, lines, bands or stains blue, violet, green or turquoise; these patterns representing > 20% of the total wing area.

5. *White & black*: dark wing area with white wide bands or stains that represent 30% - 70% of the total wing area.

6. *Orange/red & black*: wing area black, blackish or brown dark with patterns of points, lines and bands orange or red representing < 30% of the total wing area.

7. *Yellow & black*: wing area black or blackish with patterns of points, lines or bands yellow, representing < 50% of the total wing area.

8. *Yellow/white*: wing area almost entirely yellow or white, with < 10% of the wing area represented by dark patterns.

9. *Orange*: > 50% of the total wing area is colored bright orange or reddish.

These categories are based on the macroscopic appearance of the coloration patterns observable by the human eye on the dorsal surface of the wings, excluding elements reflecting ultra-violet coloration. For sexually dimorphic species, we considered only the brightest or most striking coloration of the two morphs for assignment. For species with wings colored by two or more colors, we considered the brightest or most striking color for assignment. S2 Fig. provides examples for each of the above categories of wing color.

**5, Fore wing length.** It was used as proxy for dispersal ability and either compiled from [54,55] or measured in mm from pictures in [56] or from specimens collected on BCI.

## Additional references

97. Aiello A (1980) Life history of *Dismorphia amphiona beroe* (Lepidoptera: Pieridae: Dismorphiinae) in panama. Psyche, 87: 171-175.

98. DeVries PJ, Chacon IA, Murray D (1992) Toward a better understanding of host use and biodiversity in riodinid butterflies (Lepidoptera). J Res Lep 31:103-126.

99. Duarte M, Robbins RK (2010) Description and phylogenetic analysis of the Calycopidina (Lepidoptera, Lycaenidae, Theclinae, Eumaeini): a subtribe of detritivores. Rev Bras Entomol 54: 45–65.

100. Frank JH, Lounibos LP (2009) Insects and allies associated with bromeliads: a review. Terr Arthropod Rev 1: 125–153.

101. Janzen DH, Sharkey MJ, Burns JM (1998) Parasitization biology of a new species of Braconidae (Hymenoptera) feeding on larvae of Costa Rican dry forest skippers (Lepidoptera: Hesperiidae: Pyrginae). Trop Lep 9 (Suppl. 2): 33-41.

102. Murillo-Hiller LR (2009) Early stages and natural history of *Cithaerlas p. pireta* (Satyrinae) from Costa Rica. J Lep Soc 63: 169-172.

103. Murillo-Hiller LR (2013) Early Stages and Natural History of *Mimoides pausanias prasinus* and *M. euryleon clusoculis* (Lepidoptera, Papilionidae) from Costa Rica. Ann Rev Res Biol 3: 22-30.

104. Nishida K, Nakamura I, Morales CO (2009) Plants and butterflies of a small urban preserve in the Central Valley of Costa Rica. Rev Biol Trop 57 (Suppl. 1): 31-67.

105. Peña C, Espeland M (2013) Diversity dynamics in Nymphalidae butterflies: Effect of phylogenetic uncertainty on diversification rate shift estimates. Cornell University, USA. Archive [arXiv:1302.6294](../../../../abs/1302.6294) [q-bio.PE]. <http://arxiv.org/abs/1302.6294>. Accessed 18 December 2014.

106. Penz CM (2008) Phylogenetic revision of *Eryphanis* Boisduval, with a description of a new species from Ecuador (Lepidoptera, Nymphalidae). Ins Mundi 35: 1-25.

107. Pulido HW, Andrade MG, Peñ C, Lamas G (2011) Two new taxa of *Euptychia* Hübner, 1818 (Lepidoptera: Nymphalidae: Satyrinae) from the Andes of Colombia and Peru. Zootaxa 2906: 43–51.

108. Raguso RA (1993) Preliminary checklist and field observations of the butterflies of the Maquipucuna Field Station (Pichincha Province, Ecuador). Journal Res Lep 32:135-161.

109. Robinson GS, Ackery PR, Kitching IJ, Beccaloni GW, Hernández LM (2010) HOSTS - A Database of the World's Lepidopteran Hostplants. Natural History Museum, London. <http://www.nhm.ac.uk/hosts>. Accessed: 18 December 2014.

110. Savela M (1997) Lepidoptera and some other life form. <http://www.nic.funet.fi/pub/sci/bio/life/intro.html>. Accessed 18 December 2014.

111. Sermeño-Chicas JM, Robbins RK, Lamas G, & Gámez-Alas JA (2013) Laboratory breeding of the “Living Jewels” of El Salvador, *Evenus regalis* (Cramer) and *E. batesii* (Hewitson) (Lepidoptera: Lycaenidae). Bioma enero 2013: 44-48.

112. Silva NAP, Duarte M, Diniz IR, Morais HC (2011) Host plants of Lycaenidae on inflorescences in the central Brazilian cerrado. J Res Lep 44: 95-105.

113. Willmott KR, Mallet J (2004) Correlations between adult mimicry and larval host plants in ithomiine Butterflies. Proc R Soc Lond B (Suppl.) 271: S266–S269.

114. Burd M (1994) Butterfly wing colour patterns and flying heights in the seasonally wet forest of Barro Colorado Island, Panama. J Trop Ecol 10: 601-610.
